# Impaired renal function is a major determinant of left ventricular diastolic dysfunction: assessment by stress myocardial perfusion imaging

**DOI:** 10.1007/s12149-013-0739-z

**Published:** 2013-05-28

**Authors:** Wakana Sato, Toshimitsu Kosaka, Takashi Koyama, Masaru Ishida, Kenji Iino, Hiroyuki Watanabe, Hiroshi Ito

**Affiliations:** Department of Cardiovascular and Respiratory Medicine, Akita University Graduate School of Medicine, 1-1-1 Hondo, Akita, 010-8543 Japan

**Keywords:** Chronic kidney disease, Estimated glomerular filtration rate, Coronary artery disease, Left ventricular diastolic function, Gated SPECT

## Abstract

**Objective:**

Relationships between myocardial scintigraphic parameters and renal function have not been fully determined. We investigated correlations between estimated glomerular filtration rate (eGFR) and left ventricular (LV) diastolic function using stress electrocardiographic (ECG)-gated myocardial single photon emission computed tomography (SPECT).

**Methods:**

We enrolled 136 consecutive patients with suspected coronary artery disease (CAD) who were assessed using technetium-99m stress ECG-gated myocardial SPECT. We evaluated SPECT images using 17-segment defect scores graded on a 5-point scale, summed stress score, summed rest score and summed difference score (SDS). The parameters for assessing LV diastolic function were peak filling rate (PFR), 1/3 mean filling rate and time to peak filling. The CAD was defined as SDS ≥2. Chronic kidney disease (CKD) was defined as eGFR <60 mL/min/1.73 m^2^. Patients were assigned to the following four groups (no CAD/no CKD: control group, *n* = 68; CAD/no CKD: CAD group, *n* = 24; no CAD/CKD: CKD group, *n* = 34; CAD/CKD: CAD + CKD group, *n* = 10).

**Results:**

The PFR was significantly impaired after stress in the CKD and CAD + CKD groups compared with controls (*p* < 0.001 for both). Furthermore, PFR at rest positively correlated with eGFR (*r* = 0.29, *p* < 0.001) and inversely correlated with SDS (*r* = −0.18, *p* < 0.05). Multivariate stepwise regression analysis independently associated eGFR with PFR (*β* coefficient = 0.260, *p* = 0.002).

**Conclusions:**

Our data suggest that impaired renal function is a significant determinant of LV diastolic dysfunction in patients with suspected CAD.

## Introduction

Impaired renal function confers a higher risk of coronary artery disease (CAD) and/or chronic heart failure (CHF), independently of conventional cardiovascular risk factors in patients with chronic kidney disease (CKD) [1]. Left ventricular (LV) diastolic dysfunction frequently arises in CHF patients with or without CKD [2, 3]. Moreover, LV diastolic dysfunction is a sensitive marker of myocardial ischemia and it persists longer than systolic dysfunction after release from ischemia [4]. Although an estimated glomerular filtration rate (eGFR) has been recommended to define renal dysfunction in hypertensive and diabetic patients, its clinical usefulness has not been fully recognized. Several recent clinical investigations have evaluated the relationship between renal dysfunction estimated by eGFR and cardiac functional alterations [5–7].

Electrocardiography (ECG)-gated myocardial single photon emission computed tomography (SPECT) with exercise or pharmacological stress can detect myocardial ischemia and abnormal global LV wall motion. Recent developments in quantitative gated SPECT (QGS) software allow quantitative measurement of LV diastolic function using 16-frame ECG-gated SPECT [8]. Although echocardiographic studies have indicated that LV dysfunction is relatively common in patients with CKD [9], the relationships between myocardial ischemia and renal function have not been fully examined. Therefore, we investigated the impact of myocardial ischemia and renal function on LV diastolic function by using stress ECG-gated SPECT and eGFR parameters in patients with suspected CAD.

## Materials and methods

### Study population

**Fig. 1:**
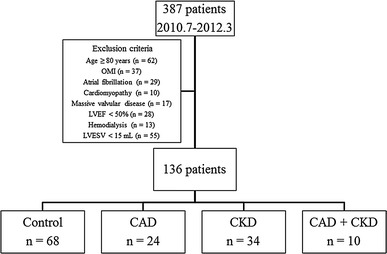
Flow chart of study population. *CAD* coronary artery disease, *CKD* chronic kidney disease, *LVEF* left ventricular ejection fraction, *OMI* old myocardial infarction

This prospective study included 387 consecutive patients with suspected CAD who had undergone stress ECG-gated myocardial SPECT at Akita University Hospital between July 2010 and March 2012. Patients older than 80 years of age were excluded. Moreover, we did not enroll patients with atrial fibrillation, major cardiovascular complications involving old myocardial infarction, idiopathic cardiomyopathy, significant valvular heart disease, depressed LV ejection fraction (EF) (<50 %) and hemodialysis. Patients with post-examination LV end-systolic volume (ESV) of <15 mL determined by QGS were also excluded to rule out patients with small hearts [10]. The Ethics Committees of Akita University Graduate School of Medicine approved the study protocol and 136 eligible patients provided written informed consent to participate in the study (Fig. 1).

### Stress and imaging protocol

Stress-rest myocardial SPECT imaging using technetium-99m (^99m^Tc) sestamibi or tetrofosmin proceeded using the 1-day protocol. A symptom-limited bicycle ergometer exercise test using the standard protocol with 12-lead ECG recordings being taken every minute was started in 65 % of patients. The ergometer exercise test was considered adequate if the patient reached >85 % of maximal predicted heart rate and/or developed chest pain. At near-maximal exercise, a 296–370 MBq dose of ^99m^Tc sestamibi or tetrofosmin was injected, and exercise continued for 1 min thereafter. Images were acquired from 30 min after isotope administration. Whenever possible, beta-blockers, calcium channel blockers, and caffeine products were discontinued 24 h before testing.

Forty-seven patients (35 % of the study population) that were deemed unable to tolerate the ergometer test or to achieve 85 % of the maximal predicted heart rate (HR) underwent a pharmacological stress test using the adenosine infusion protocol. Adenosine (0.120 mg/kg/min) was infused using an automatic pump over a period of 6 min and then ^99m^Tc sestamibi or tetrofosmin was injected 3 min after starting the adenosine infusion. The patients at rest were administered 3 h later with 740 MBq of ^99m^Tc sestamibi or tetrofosmin.

End-diastolic and end-systolic myocardial perfusion SPECT images were acquired using a Symbia T2 dual-headed gamma camera (Siemens Medical Solutions, Erlagen, Germany) with circular 180° acquisition for 36 projections at 20 s per projection and a low-energy, general purpose collimator. A 20 % window was centered on the 140-keV peak for ^99m^Tc tracers during the imaging process and the maximal matrix size was 64 × 64. The R–R interval was divided by the R wave trigger into 16 equal portions while acquiring ECG-gated images.

### Myocardial SPECT and ECG-gated parameters

We reconstructed the SPECT imaging data using a data processor combined with filtered back-projection. Summed stress (SSS), rest (SRS), and difference (SDS) scores were semi-quantified from the SPECT data according to a 17-segment, 5-point scoring system using automated Heart Score View software (Nihon Medi-Physics Co Ltd, Tokyo, Japan) [11, 12]. SDS of ≥2 indicated significant inducible myocardial ischemia.

Reconstructed short-axis ECG-gated stress and rest SPECT images were processed using the QGS software to automatically calculate LV end-diastolic volume (EDV), LVESV and LV ejection fraction. The LV time–volume curve was plotted from each of the 16 frames, and generated using Fourier transforms. From this time–volume curve and its first differentiated curve which was transformed into a LV time-filling rate (d*V*/d*t*) curve, we obtained peak filling rate (PFR), mean filling rate during the first-third of diastole (1/3MFR) and time to peak filling (TPF) and used them to assess global LV diastolic function. We defined PFR as the greatest filling rate at early diastole and it corresponded to the first maximal d*V*/d*t* value normalized to EDV, and defined TPF as the interval from the time at end systole to that at PFR.

### Evaluation of renal function

The glomerular filtration rate was estimated from the equation for Japanese patients as follows: eGFR = 194 × (serum creatinine)^−1.094^ × age^−0.287^ × (0.739 if female) [13]. Forty-four (32 %) of our patients had CKD defined according to the National Kidney foundation definition as eGFR <60 mL/min/1.73 m^2^. Serum creatinine used to calculate eGFR was obtained within 32 ± 19 days from the time of myocardial SPECT.

### Echocardiography

Echocardiography proceeded using an iE33 cardiac ultrasound unit (Phillips Medical Systems, Bothell, WA, USA). Both LVEF and LV mass index (LVMI) were calculated from the standard M-mode echocardiograms. Mitral inflow velocity was traced and we derived peak early (*E*) and late (*A*) transmitral flow velocities, the ratio of early to late peak velocities (*E*/*A*) and the deceleration time of *E* velocity [14, 15]. Early peak diastolic annular velocity (*E*′) was determined from spectral pulsed-wave tissue Doppler imaging (TDI) recordings and the mitral *E*/*E*′ ratio was calculated. The sample volume at the septal corner of the mitral annulus was used for the apical-four chamber view [16–19].

### Assignment of patients to groups

We assigned patients to groups based on the presence or absence of CAD and CKD as follows: neither CAD nor CKD (controls, *n* = 68), CAD alone (CAD, *n* = 24), CKD alone (CKD, *n* = 34), and both CAD and CKD (CAD + CKD, *n* = 10; Fig. 1).

### Statistical analysis

Continuous variables are expressed as mean ± SD and categorical variables are expressed as numbers (%). Differences among groups were analyzed by one-way analysis of variance followed by a post hoc Tukey–Kramer test for multiple comparisons. Categorical variables were analyzed using the *χ* ^2^ test. Left ventricular function at stress and rest were compared using Student’s paired *t* test and correlations between two variables were determined using Spearman’s rank correlation. Predictors of PFR were assessed using multivariate regression analysis with stepwise selection. Statistical significance was indicated at *p* < 0.05. All of these data were statistically analyzed using SPSS version 19.0 (SPSS Inc., Chicago, IL, USA).

## Results

### Clinical characteristics of the patients

Table 1 shows the characteristics of the patients (mean age 65.6 ± 11.3 years, male 76 %). Age, gender, body mass index, systolic and diastolic BP, HR did not significantly differ among the four groups. Dyslipidemia was more prevalent in the CAD + CKD group than in any other group. The prevalence of hypertension and diabetes mellitus was predominant in the CAD + CKD group, although there was no significant difference among the four groups.

**Table 1:** Characteristics of the patients

	Control	CAD	CKD	CAD + CKD	*p*
	(*n* = 68)	(*n* = 24)	(*n* = 34)	(*n* = 10)	
Age (years)	65 ± 12	64 ± 13	68 ± 10	66 ± 12	ns
Sex (male)	52 (76 %)	19 (79 %)	23 (68 %)	9 (90 %)	ns
BMI (kg/m^2^)	23.8 ± 3.9	23.8 ± 5.2	25.3 ± 4.8	26.0 ± 4.2	ns
SBP (mmHg)	143 ± 24	142 ± 22	145 ± 21	152 ± 24	ns
DBP (mmHg)	77 ± 12	79 ± 19	74 ± 14	71 ± 23	ns
HR (beats/min)	66 ± 9	67 ± 10	69 ± 11	62 ± 13	ns
Hypertension	42 (62 %)	18 (75 %)	28 (82 %)	9 (90 %)	ns
Dyslipidemia	33 (49 %)	13 (54 %)	19 (56 %)	10 (100 %)	<0.05
Diabetes mellitus	26 (38 %)	8 (33 %)	17 (50 %)	8 (80 %)	ns

Data are expressed as mean values ± SD or numbers (%)

*BMI* body mass index, *DBP* diastolic blood pressure, *HR* heart rate, *SBP* systolic blood pressure

### Echocardiographic and biochemical findings

Table 2 shows the echocardiographic and biochemical parameters. The four groups had similar LVMI, LVEF, and *E*/*A* values, whereas *E*/*E*′ was significantly higher in the CKD and CAD + CKD groups than the control group. *E*′ tended to decrease in the CKD and CAD + CKD groups, although the difference did not reach statistical significance. Only high-density lipoprotein cholesterol, serum creatinine and uric acid significantly differed among the biochemical parameters.

**Table 2:** Echocardiographic variables and biochemical parameters

	Control	CAD	CKD	CAD + CKD	*p*
	(*n* = 68)	(*n* = 24)	(*n* = 34)	(*n* = 10)	
LVMI (g/m^2^)	121 ± 35	132 ± 37	139 ± 36	137 ± 30	ns
*E*/*A*	0.88 ± 0.32	0.90 ± 0.32	0.82 ± 0.45	0.93 ± 0.33	ns
Deceleration time (ms)	211 ± 56	185 ± 42	243 ± 86^a^	201 ± 70	<0.05
*E*′ (cm/s)	8.0 ± 3.1	7.9 ± 2.5	6.8 ± 2.4	5.5 ± 1.1	ns
*E*/*E*′	8.1 ± 3.1	8.1 ± 2.3	10.3 ± 5.5^b^	11.6 ± 5.3^c,d^	<0.05
LVEF (%)	66 ± 6	65 ± 9	67 ± 8	61 ± 15	ns
Hemoglobin (g/dL)	13.0 ± 1.9	13.6 ± 2.0	12.6 ± 2.0	12.0 ± 2.6	ns
Total cholesterol (mg/dL)	175 ± 37	191 ± 48	174 ± 39	177 ± 66	ns
Triglycerides (mg/dL)	129 ± 63	141 ± 78	155 ± 78	173 ± 80	ns
HDLC (mg/dL)	57 ± 19	52 ± 14	48 ± 14	38 ± 15*	<0.01
LDLC (mg/dL)	101 ± 28	111 ± 37	102 ± 35	126 ± 51	ns
HbA1c (%)	62 ± 1.1	6.1 ± 1.0	6.5 ± 1.5	6.9 ± 1.6	ns
Serum creatinine (mg/dL)	0.71 ± 0.14	0.70 ± 0.14	1.62 ± 1.32^a,b^	1.75 ± 0.46^c,d^	<0.001
Uric acid (mg/dL)	5.2 ± 1.3	5.4 ± 1.3	6.5 ± 1.4^b^	7.3 ± 2.6^c,d^	<0.001

Data are expressed as mean values ± SD

*E* early diastole velocity of mitral annulus, *E*/*A* ratio of mitral *E* and *A* velocities, *E*/*E*′ ratio of mitral *E* and *E*′, *HbAlc* hemoglobin A1c, *HDLC* high-density lipoprotein cholesterol, *LDLC* low-density lipoprotein cholesterol, *LVMI* left ventricular mass index

^a^CAD vs. CKD all *p* < 0 05

^b^Control vs. CKD

^c^Control vs. CAD + CKD

^d^CAD vs. CAD + CKD

### Global left ventricular functional analysis

Table 3 shows the results of the global LV functional analysis. The EDV after stress and at rest did not significantly differ among the four groups. Post-stress ESV was significantly greater in the CAD group and in the CKD group at rest than in the control group. Compared with LV function at rest, LVEF was significantly decreased after stress in the CAD and CAD + CKD groups. Post-stress PFR was significantly decreased in the CKD and CAD + CKD groups compared with the control group, and PFR at rest was significantly decreased in the CKD group as compared with the control and CAD groups. The 1/3MFR after stress and at rest was decreased only in the CKD group. The TPF did not significantly differ among the four groups and LV diastolic functional parameters did not significantly differ between the CAD and control groups.

**Table 3:** Radionuclide imaging variables

	Control (*n* = 68)	CAD (*n* = 24)	CKD (*n* = 34)	CAD + CKD (*n* = 10)	*p*
EDV (mL)					
Stress	79 ± 28	90 ± 29	83 ± 32	92 ± 27	ns
Rest	78 ± 27	90 ± 26	89 ± 42	90 ± 29	ns
ESV (mL)					
Stress	29 ± 15	41 ± 19^a^	34 ± 19	46 ± 17	<0.01
Rest	26 ± 15	36 ± 17	37 ± 28^b^	38 ± 16	<0.05
LVEF (%)					
Stress	64 ± 8	56 ± 10^a^	60 ± 10	52 ± 9^c^	<0.001
Rest	69 ± 9	62 ± 9	62 ± 12	60 ± 8	ns
PFR (EDV/s)					
Stress	2.30 ± 0.56	2.00 ± 0.44	1.90 ± 0.61^b^	1.67 ± 0.53^c^	<0.001
Rest	2.34 ± 0.60	2.22 ± 0.54	1.79 ± 0.57^b,d^	1.96 ± 0.43	<0.001
1/3MFR (EDV/s)					
Stress	1.25 ± 0.38	1.02 ± 0.43	0.96 ± 0.33^b^	0.93 ± 0.42	<0.001
Rest	1.31 ± 0.36	1.23 ± 0.26	1.04 ± 0.35^b^	1.14 ± 0.36	<0.01
TPF (ms)					
Stress	177 ± 41	203 ± 69	185 ± 66	211 ± 84	ns
Rest	180 ± 38	174 ± 31	180 ± 65	170 ± 44	ns

Data are expressed as mean values ± SD

*EDV* end-diastolic volume, *ESV* end-systolic volume, *LVEF* left ventricular ejection fraction, *PFR* peak filling rate, *1/3MFR* 1/3 mean filling rate, *TPF* time to peak filling

^a^Control vs. CAD: all *p* < 0 05

^b^Control vs. CKD

^c^Control vs. CAD + CKD

^d^CAD vs. CKD

**Fig. 2:**
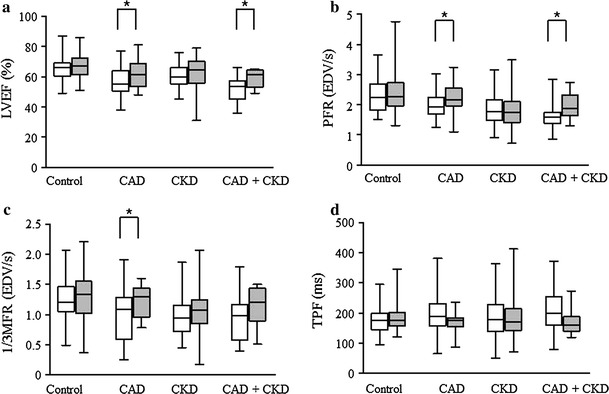
Comparison of global left ventricular function between stress (*open bars*) and rest (*shaded bars*). **a** Left ventricular ejection fraction (LVEF). **b** Peak filling rate (PFR). **c** One-third of mean filling rate (1/3MFR). **d** Time to peak filling (TPF). Compared with global functional analysis at rest, LVEF and PFR significantly decreased in CAD and CAD + CKD groups after exercise stress or adenosine loading. *CAD* coronary artery disease, *CKD* chronic kidney disease. Data are expressed as mean ± SD. *p < 0.05

Both LVEF and PFR significantly decreased after exercise stress or adenosine loading compared with global LV function at rest in the CAD and CAD + CKD groups, whereas TPF tended to be prolonged in these groups without reaching statistical significance (Fig. 2).

### Assessment of factors related to PFR

**Fig. 3:**
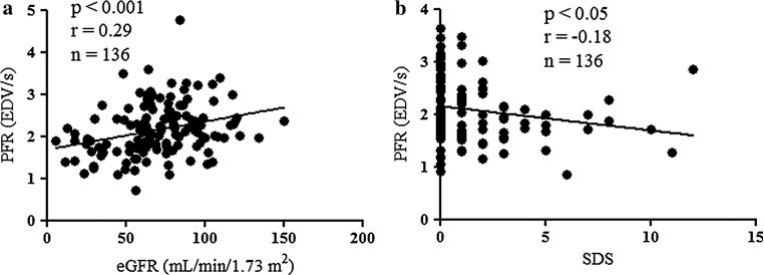
Correlation between peak filling rate (PFR) and estimated glomerular filtration rate (eGFR) (**a**), summed difference score (SDS) (**b**). The eGFR correlated significantly and positively with PFR at rest in all 136 patients (**a**) and a weak inverse correlation between SDS and PFR after stress was nevertheless significant (**b**). *EDV* end-diastolic volume

The eGFR correlated significantly and positively with PFR at rest in all 136 patients (Fig. 3a) and a weak inverse correlation between SDS and PFR was nevertheless significant (Fig. 3b). We analyzed which clinical and scintigraphic parameters were independently associated with PFR after stress by including age, eGFR, SDS, HR, LVMI, hemoglobin, and LVEF in a multivariate stepwise regression model. The results independently associated age, eGFR, and SDS with PFR, but not LVMI, hemoglobin and LVEF (Table 4).

**Table 4:** Multivariate stepwise regression analysis of peak filling rate and related parameters

	*β* coefficient	*t*	*p*
Age	−0.293	−3.630	<0.0001
eGFR	0.260	3.242	0.002
SDS	−0.193	−2.401	0.018
HR	0.118	1.442	0.152
LVMI	−0.090	−1.073	0.285
Hemoglobin	−0.034	−0.402	0.688
LVEF	0.013	0.162	0.872

*eGFR* estimated glomerular filtration rate, *HR* heart rate, *LVEF* left ventricular ejection fraction, *LVMI* left ventricular mass index, *SDS* summed difference score

## Discussion

Here, we presented data regarding the relationship between renal dysfunction determined by eGFR and LV diastolic dysfunction determined by ECG-gated myocardial SPECT in patients with suspected CAD. Nearly 25 % of our patients referred for ECG-gated SPECT had results indicating CAD, and almost 30 % had CKD. These patients had a higher prevalence of LV diastolic dysfunction than those without CAD and CKD. On the other hand, LV systolic dysfunction was evident in patients who developed myocardial ischemia after stress. From the different specificities among parameters for determining LV diastolic function, we selected PFR for multivariate analysis, because it has been considered the most sensitive parameter which represents overall LV diastolic function [20]. The results of the multivariate regression analysis showed that rather than the severity of myocardial ischemia (SDS), eGFR was more closely related to LV diastolic dysfunction estimated as PFR.

Echocardiographic investigations using conventional and pulsed-wave TDI have revealed abnormal LV diastolic function in patients with CKD [9]. Since CKD is more likely to be complicated by CAD, the simultaneous evaluation of both renal function and myocardial ischemia is important in consideration of LV diastolic function. Although cardiac performance in patients with heart disease has been widely assessed using echocardiography, myocardial ischemia cannot be precisely detected by conventional means. We prefer ECG-gated SPECT because it offers distinct advantages over echocardiography in terms of quantifying myocardial ischemia, and LV function in that data are collected over hundreds of cardiac cycles, and operator-independent parameters are provided essentially automatically. With respect to LV diastolic function, recent studies have revealed close relationships between ECG-gated radionuclide ventriculographic findings and SPECT indices such as PFR, 1/3MFR, and TPF [21, 22]. Furthermore, these parameters derived from ECG-gated SPECT that closely correlate with LV end-diastolic pressure at subsequent cardiac catheterization [20]. Thus, while the use of ECG-gated SPECT is limited, this modality is non-invasive and useful for assessing LV diastolic function. We found that PFR and 1/3MFR were decreased in patients with CKD, but TPF was not significantly prolonged. Both PFR and TPF reflect the integrity of isovolumic relaxation and subsequent LV filling, these discrepant findings may be partially explained by the lower temporal resolution of 16-frame data, which might not be enough for providing adequate TPF values. Left ventricular systolic dysfunction is frequently associated with severe CAD and it is a major determinant of prognosis [23]. Post-ischemic regional LV wall motion abnormalities during systole continue only for 15–30 min after exercise stress, whereas LV diastolic dysfunction persists far longer [4]. Myocardial SPECT imaging precisely determined myocardial ischemia and post-ischemic LV stunning in the present study, but no significant differences among LV diastolic functional parameters were confirmed in patients with CAD (Table 3). Moreover, SDS was not as closely correlated (Fig. 3b) and it was independently associated with PFR. These findings suggested that LV diastolic dysfunction persisted in CKD patients regardless of myocardial ischemia.

Several pathophysiological conditions such as LV hypertrophy, CAD, microvascular abnormalities, interstitial fibrosis, altered fluid and electrolyte metabolism and neurohumoral alterations might contribute to the mechanisms of LV diastolic dysfunction in patients with CKD. Our clinical study cannot explain the different roles played by all of these factors. Left ventricular hypertrophy is one potential cause of LV diastolic dysfunction in patients with CKD, yet LVMI did not significantly differ among our study groups, making any significant contribution of its structural effect on LV function unlikely. One possible mechanism associated with CKD such as over-activation of the rennin–angiotensin–aldosterone system (RAAS) might help to explain our findings. In fact, the RAAS plays a key role among profibrotic factors [24, 25]. Furthermore, mild CKD results in early cardiac fibrosis with mild LV diastolic impairment and preserved systolic function [26]. Identifying the relative contributions of the profibrotic effect on LV diastolic dysfunction by measuring markers of collagen turnover might provide us further information about the pathogenesis of cardiac functional alterations with CKD patients.

### Limitations

This study has several important limitations. The small study cohort might have blunted between-group differences, especially those regarding possible additional influences of the duration of renal dysfunction, hypertension and diabetes on LV diastolic dysfunction. It is possible that LV diastolic functional parameters such as PFR and 1/3MFR might not show significant differences between the CAD and control groups because of an imbalanced inter-group population. Thus, our results and conclusions can only be regarded as preliminary. We have no information about albuminuria for the patients in whom eGFR was ≥60 mL/min/1.73 m^2^. Therefore, some patients included in the control group might in fact have had CKD. The principal purpose of our study was to examine relationships between eGFR and scintigraphic findings in patients with various degrees of renal function. Further studies are needed to determine associations between albuminuria and scintigraphic parameters in patients with CKD. Previous studies of post-stress myocardial dysfunction focused on patients with exercise-induced ischemia, however, some reports have demonstrated post-ischemic stunning after vasodilator stress [23, 27–29]. Myocardial perfusion imaging with adenosine relies on including relative flow heterogeneity in myocardial regions supplied by normal and diseased coronary arteries, and in some patients with significant stenosis, adenosine may induce true ischemia as a result of coronary steal [30]. Although 35 % of our study population underwent a pharmacological stress test, we cannot neglect the possibility of the occurrence of post-ischemic stunning after adenosine stress. Finally, ECG-gated myocardial SPECT permits only an indirect estimate of diastolic function relative to LV filling dynamics, whereas the relationships in LV diastolic performance could not be precisely evaluated because LV pressure–volume was not simultaneously measured. Nevertheless, considering the absence of significant differences among the four groups in age, BP, HR and LVEF, which greatly influence filling parameters, our study yielded valuable information about the relationship between renal function and LV diastolic function.

## Conclusion

Left ventricular diastolic dysfunction is more closely associated with eGFR than with myocardial perfusion abnormalities in patients with suspected CAD. Therefore, impaired renal function might predict LV diastolic dysfunction determined by ECG-gated myocardial SPECT more effectively than myocardial ischemia.
